# Artificial Intelligence as an Add‐On Instrument in Fetal Ultrasound; Sonographers' and Obstetricians' Expectations

**DOI:** 10.1002/pd.70133

**Published:** 2026-03-30

**Authors:** Renée M. Smit, Chiara C. M. M. Lap, Rima Arnaout, Anita J. Moon‐Grady, Monique C. Haak

**Affiliations:** ^1^ Department of Obstetrics and Gynecology Division of Fetal Medicine Leiden University Medical Center Leiden the Netherlands; ^2^ Department of Medicine Division of Cardiology University of California San Francisco San Francisco California USA; ^3^ Department of Pediatrics Division of Cardiology University of California San Francisco San Francisco California USA

**Keywords:** artificial intelligence, automatic image analysis, fetal ultrasound, sonographers, survey

## Abstract

**Objective:**

Artificial intelligence applications (AIA) in fetal ultrasound are rapidly evolving, yet their integration into routine clinical practice remains limited. This study explores the attitudes, expectations and concerns of obstetric sonographers and gynecologists regarding AIA as a diagnostic aid during fetal anomaly scans.

**Method:**

An online survey was distributed to sonographers, midwives, gynecologists and fetal medicine consultants performing screening and diagnostic fetal ultrasounds in the Netherlands. The survey included demographics, current use of artificial intelligence (AI), and 22 attitudinal statements rated on acceptance, efficiency, safety, and quality. Respondents were grouped by age, AI experience, and scan type.

**Results:**

Among 151 respondents, 47% were sonographers performing standard anomaly screening and 49% were performing diagnostic anomaly scans at tertiary‐level centers. AI was already used by 62%, primarily for automated biometry. Overall, 87% supported AIA use, yet 86% emphasized retaining final clinical authority. Concerns about data security (10%) and software manipulation (12%) were minimal. While 73% of the surveyed respondents believed AIA can improve scan completeness, only 39% expect workload reduction and 70% expect improved anomaly detection.

**Conclusion:**

Obstetric sonographers generally support AIA integration, anticipating a positive impact on screening outcomes. However, human decision‐making remains essential. These insights may guide future implementation strategies and offer momentum to influence referral practices.

## Summary

- What's already known about this topic?
  ◦ Artificial intelligence applications in fetal ultrasound are advancing at a tremendous rate, yet their integration into routine clinical practice remains limited.
- What does this study add?
  ◦ Provides a first insight into how obstetric sonographers view AI‐assistance in their work.
  ◦ Identifies strong support for AI with a parallel desire to maintain control over clinical decisions.

## 1 Introduction

In recent years artificial intelligence (AI) has rapidly advanced, offering new possibilities for improving the accuracy and efficiency of fetal ultrasound imaging [1, 2].

Systems that enable the identification and storage of planes, track the completeness of a scan, or automatically measure certain structures are already commercially available. Algorithms that aim to recognize abnormalities in the planes retrieved in the setting of a standard anomaly scan have been developed [3, 4, 5, 6]. This will lead to an AI‐driven scanning assistant, which alerts sonographers to a possible malformations with the need to refer a pregnant woman to a center for prenatal diagnosis.

To fully harness the potential of AI systems, clinicians must actively collaborate with these tools. However, risks arise from both over‐reliance and reluctance to use AI assistance. It is essential for clinicians to discern when to trust and when to reject AI outputs in order to successfully implement these AI systems in clinical practice [7, 8].

In the field of radiology, AI is already accepted as an assistive tool in daily work for certain use cases. In the process of implementing these AI tools, the most common enablers for the acceptance were the expectancy of potential added value, to decrease errors in diagnosis and to increase efficiency and quality in patient care [9, 10]. Ultrasound applications for AI tools have been adopted more slowly, in part because of the heterogeneous nature of ultrasound image quality and applications and differences in practice patterns worldwide. Outside the United States, for example, fetal ultrasound is performed by a combination of gynecologists (in training), Maternal Fetal Medicine (MFM) specialists, doctors working as sonographers in a tertiary fetal medicine unit, sonography practitioners working in midwifery practices and midwives (collectively “obstetric sonographers”). Their attitudes toward automated image analysis (AIA) as an add‐on diagnostic tool in fetal ultrasound are not yet studied but are important for successful implementation in the future.

The aim of this study is to explore the opinions and expectations of obstetric sonographers regarding the use of automated image analysis as a diagnostic tool during fetal anomaly scans across various healthcare settings in the Netherlands.

## 2 Methods

An online questionnaire was designed (Castor ECD, Castor Research Inc. version 1.6). The questionnaire was divided into two sections. The first section consisted of multiple‐choice and fill‐in‐the‐blank questions to determine participants baseline characteristics (profession, age, types of ultrasound examinations performed, years of experience) and their knowledge and use of AI in daily life and at work. In the second section, twenty‐two statements regarding acceptance, efficiency, expectations, safety and quality of automated image analysis in fetal ultrasound were prompted. The participants responded on a 6‐point Likert‐type scale, ranging from “strongly disagree” to “strongly agree” or the option “I do not know”. Statements were randomly arranged and were overlapping on many items in context in order to cross‐check for consistency of the answers.

Between March and August 2024 gynecologists, maternal‐fetal medicine specialists, fetal sonographers and midwives, performing fetal anomaly scans in various primary care centers and diagnostic scans in tertiary care settings in the Netherlands were recruited by mail, social media and at a national fetal ultrasonography symposium. Respondents were informed about the purpose and context of the questionnaire as well as the anonymous use of their responses for scientific publication. After consent, the questionnaire could be opened. As no patient data were obtained, this study was waived from local medical ethical review board approval. This study complies with the Declaration of Helsinki.

### 2.1 Statistical Analysis

Descriptive statistics and frequency analysis were performed for baseline questions and presented in numbers and percentages. The 6‐point Likert‐type scale items were recoded: *strongly disagree* and *disagree* represent the disagree group. *Strongly agree* and *agree* represent the agree group. In order to apply the chi‐squared statistical test, the response “I do not know” was treated as missing when it accounted for less than 10% of the answers for a given statement. For statements with more than 10% “I do not know” responses, the item was excluded from statistical testing, and only descriptive statistics were reported to ensure reliable analysis [11].

The respondents were categorized into two groups based on age: a younger and an older group. The division was made at 48 years of age, corresponding to the median age of the overall cohort.

Furthermore, participants were categorized into subgroups based on the type of anomaly scans they perform: standard anomaly scans aligned with routine mid‐trimester fetal ultrasound examinations as defined by the International Society of Ultrasound in Obstetrics and Gynecology (ISUOG) guidelines for low‐risk pregnancies [12] conducted in various primary care settings or diagnostic scans conducted in cases with a higher risk of fetal anomalies, which in the Netherlands are exclusively performed at tertiary care centers. An additional grouping criterion was whether participants already utilized AI in any form during ultrasound.

SPSS Statistics version 29 (IBM, Armonk, NY, USA) was used. A *p*‐value ≤ 0.05 was considered significant.

## 3 Results

We received 205 responses, but in 54 surveys only baseline characteristics were available. Therefore, 151 surveys were included in the analysis, of which 146 surveys were fully complete (97%). The remaining five surveys were filled for at least 60%. The baseline characteristics of the respondents are presented in Table 1. The majority of the respondents were female (85%). Most of the respondents (85%) had more than 6 years of experience in fetal ultrasound. Of the respondents, 47% were midwives or sonographers performing standard anomaly scans in midwifery practices. Additionally, 49% were doctors (13% working as a sonographer in a tertiary fetal medicine unit, 36% MFM specialists) all performing diagnostic scans in departments of tertiary fetal medicine units of university hospitals.

**TABLE 1:** Baseline characteristics of survey participants.

		Total *n* = 151
Age	Years	48 (40–59)
Sex	Female	128 (85%)
Profession	(Midwife) sonographer	71 (47%)
	Doctor sonographer in fetal medicine unit	19 (13%)
	Maternal fetal medicine specialist	55 (36%)
	Gynecologist in training	3 (2%)
	Other	3 (2%)
Experience	0–5 years	23 (15%)
	6–15 years	51 (34%)
	> 15 years	77 (51%)
Type of scan	Screening anomaly scan	61 (40%)
	Diagnostic anomaly scan	90 (60%)
Use of AI in work^a^	Yes	94 (62%)

*Note:* Presented as median (interquartile range) or number (percentage).

^a^ The level of AI used is limited to automatic biometric measurements.

Of the respondents, 101 (62%) report the use of AIA in their daily work ranging from occasional to continuous use. Respondents who currently utilize AI tools primarily employ them for automatic measurements for fetal biometry, including head circumference, abdominal circumference, femur length, and crown‐rump length. In 65% (66/101) of the AI‐users, the frequent need for manual adjustment of the calipers was spontaneously reported in the free‐text fields. Other examples provided in the free‐text fields included the use of AI tools to monitor the completeness of the exam or to automatically annotate the imaging planes. One respondent reported using AIA to automatically generate standard cardiac planes during live scanning.

### 3.1 Acceptance

**FIGURE 1:**
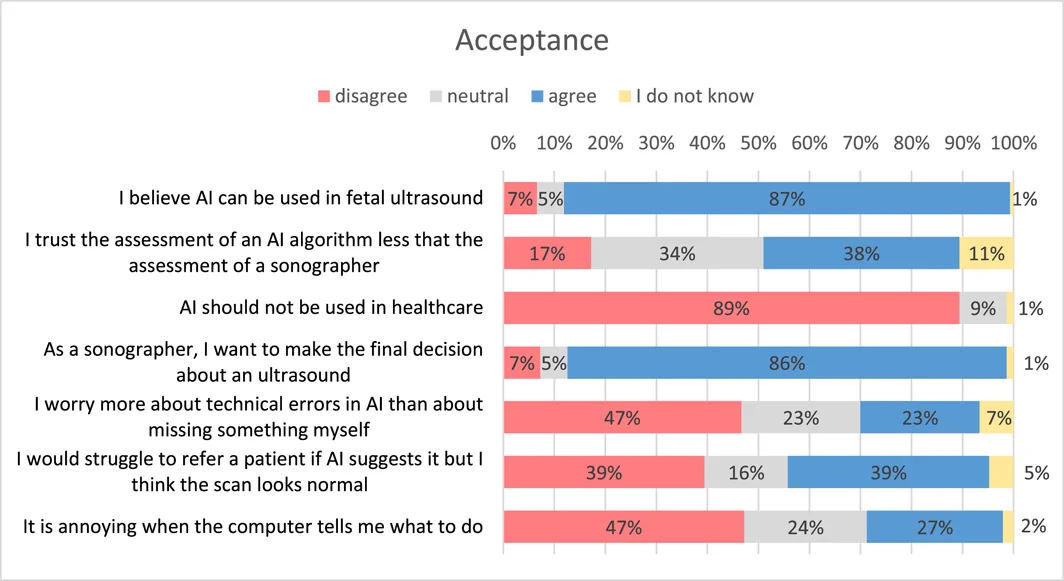
Fetal sonographers' views on the acceptance of artificial intelligence (AI). Responses are shown as percentages.

As shown in Figure 1, 87% of respondents found the use of AIA in fetal ultrasound to be acceptable. This perception is further corroborated by 89% disagreement with a statement suggesting the opposite. However, a substantial majority (86%) agreed that sonographers should retain the primary authority to interpret and finalize ultrasound findings. This perspective was consistently reinforced in the respondents' open‐ended comments. Statements about negative emotional responses to AIA, such as anxiety were inconclusive.

### 3.2 Safety and Quality

**FIGURE 2:**
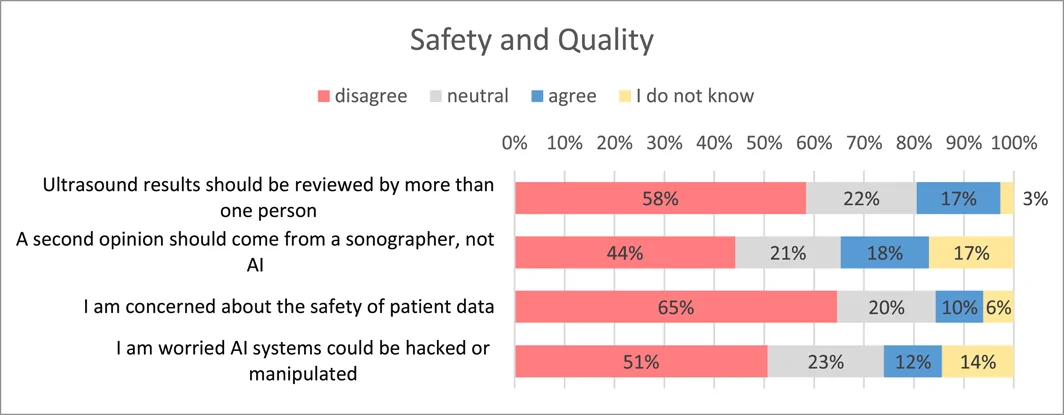
Fetal sonographers' perceptions of safety and quality of artificial intelligence (AI). Responses are displayed as percentages.

Only 10% of respondents expressed concerns regarding the security of patient data (Figure 2), while 12% of respondents expressed concerns about potential external manipulation of the AIA software. The younger age group (*n* = 72) showed to be more concerned on this subject as 16% agreed compared to 7% in the older age group (*n* = 79), however the older age group had a more neutral opinion (31% vs. 14%) or more “I do not know” (18% vs. 10%; *p* 0.017).

The majority (58%) of the respondents disagreed with the statement *’Ultrasound results should be reviewed by more than one person’* Additionally, only 17% believed that a second look of another person would be beneficial. Furthermore, 44% disagreed with the statement: *A second opinion should come from a sonographer, not AI*. These results did not differ significantly in the subgroups.

### 3.3 Efficiency

**FIGURE 3:**
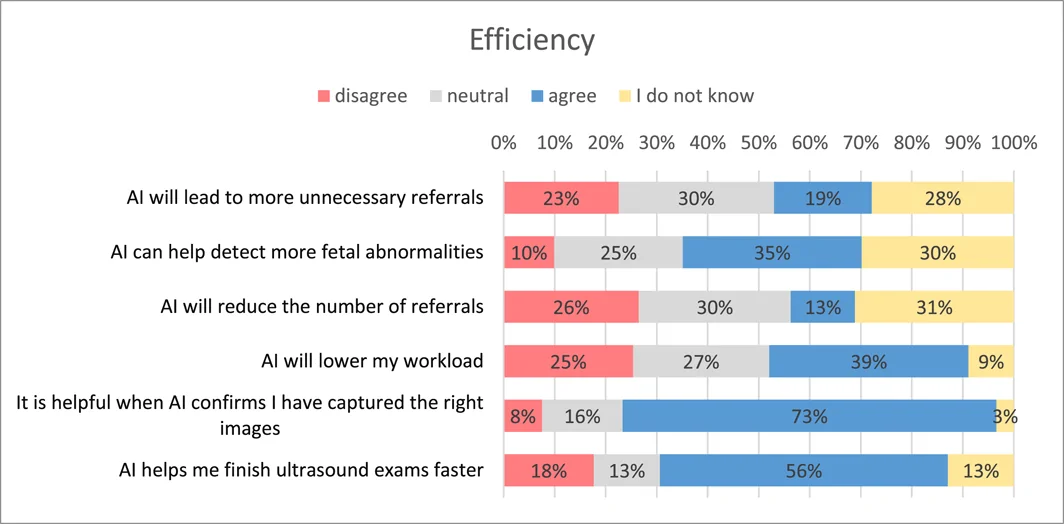
Fetal sonographers' perspectives on the efficiency of artificial intelligence (AI) in ultrasound practice. Responses are shown as percentages.

Of the respondents, 73% appreciate that AIA can be effectively used to monitor the completeness and correctness of the examination (Figure 3). When comparing age groups, this opinion is more pronounced in the younger group; 80% versus 68% (*p* = 0.019) in the older group. Although 56% thinks that the use of AIA can decrease the scanning time, only 39% of the respondents think that AIA will actually reduce the overall workload. There was no difference between the groups who performed standard versus diagnostic anomaly scans and between the groups who used or did not use AI in daily practice. Statements regarding the expected effect of AIA on referral patterns were inconclusive.

### 3.4 Expectations

**FIGURE 4:**
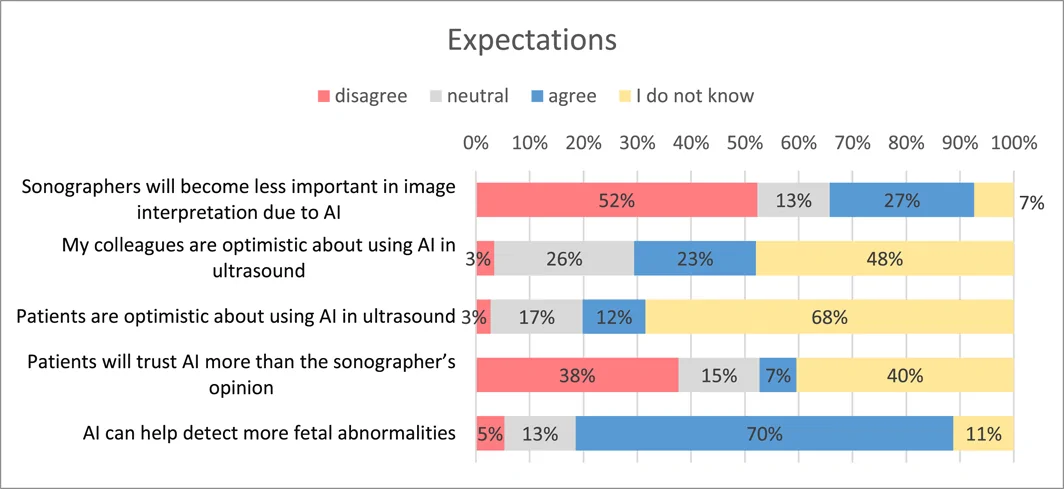
Fetal sonographers' expectations regarding the use of artificial intelligence (AI) in ultrasound practice. Responses are presented as percentages.

Seventy percent of the respondents expect that AIA will improve the detection rate of fetal abnormalities. In addition, 52% believe that the introduction of AIA will not reduce the role of the sonographer in the assessment of the anomaly scan (Figure 4). The majority of respondents do not know what the opinion of patients (68%) or their colleagues (48%) is toward the use of AIA in fetal anomaly scans. There was no significant difference when comparing the above described subgroups of respondents.

### 3.5 Final Concluding Question

A final question concerned the general opinion of the use of AIA in fetal anomaly scans. 69% expressed a positive or very positive view, only 4% felt negative about the use of AIA and none of the respondents were very negative.

## 4 Discussion

This survey among professionals in the field of obstetric ultrasound screening showed that the majority (69%) hold a positive attitude toward the incorporation of AIA into routine clinical practice. The participants expect an increase in efficiency and a reduction in scanning time as the most important advantages. Most of our participants already reported utilizing automatic fetal biometry measurements, while some employed automatic recording and annotation of images. The general expectation is that the use of AIA as a diagnostic tool in the standard anomaly scans will lead to an increased detection rate of abnormalities. There is a strong consensus that the examiner should always have the final decision on whether to refer a case for additional evaluation. Subgroups by age, type of scan, or use of AI in daily practice differed in responses only regarding data security and monitoring completeness and correctness of examinations.

The use of artificial intelligence assistance in fetal ultrasound for identifying potential anomalies has shown promising results in retrospective studies and database analyses [3]. However, algorithms with the specific aim to aid in the identification of anomalies at the time of the ultrasound examination, have not been widely integrated into routine clinical practice yet. To ensure successful integration and implementation of AIA tools in clinical settings, it is essential to consider the opinions of the end‐users, specifically sonographers, who will play a critical role in the adoption and application of these technologies in the near future [13, 14].

We found that there is a generally positive opinion toward the integration of AIA into routine fetal ultrasound clinical practice and the expectation of an increase in efficiency and a reduction in scanning time. This sentiment was also found in a systematic review conducted by Henlzer et al. that synthesized perspectives on AI in patient care, across various medical disciplines, and demonstrated that healthcare professionals generally endorse the adoption of AI technologies in clinical practice. Among the anticipated benefits, increased efficiency and thereby reduced workload were mostly reported [15].

The majority of our participants already reported utilizing automatic fetal biometry measurements. This previous experience may have contributed to the positive view toward new AIA tools, since clinicians with prior exposure to AI tools are more likely to integrate AI in medical practice as reported by Henry et al. in a qualitative study [16].

Interestingly, 65% of the respondents who used automatic fetal biometry measurements reported that they felt the urge to manually adjust the calipers. We speculate that adjustment of automatic measurements by the sonographer might be a reflection of their strong need to have that final say on interpretation of findings, as 86% of the responders agreed with the statement *As a sonographer, I want to make the final decision about an ultrasound*. Although this survey cannot answer the question if a clinically relevant adjustment of the calipers is always needed or that the sonographer feels the urge to manually adjust them to feel in control, the answer on this statement suggests that it may be more psychologically driven than actually needed. In fact, in a recent study by He et al. [17] using AIA for left ventricular function assessment in adult echocardiography, physicians were blinded to whether a sonographer or an AI tool made the measurements and adjusted the measurements less often (16.8%) when the AI tool made the measurement than when the sonographer did.

The wish to have an AI tool that acts as a clinical decision support tool and keeps the final clinical decision to the healthcare professional was also found in survey studies in oncology imaging [15, 18]. This final say does not appear to stem from a lack of trust in the technology—often cited as a barrier to the implementation of AI in clinical practice [9, 10]. In fact, 70% of respondents agreed on the potential usefulness of AIA for increasing the detection rate of fetal abnormalities. In contrast, a standard double‐look by a colleague was considered beneficial by only 17% of respondents, suggesting that a significant portion may already perceive AI as a more valuable second opinion than a human reviewer. This aligns with findings from a previously published qualitative interview study among Dutch obstetricians, which explored expectations of AIA in clinical practice and concluded that participants hoped AIA would outperform them in diagnostic accuracy [19]. The effect of double‐look by another sonographer in fetal anomaly scanning has not been studied, but such an approach will be very time consuming and thereby not practical and expensive. It may also introduce a sense of vulnerability through perceived surveillance or professional scrutiny. In radiology double‐reading is more common, for example in mammography screening programs [20]. Detection and recall rates with double reading were comparable to AI‐supported screen reading, but AIA showed a substantially lower screen‐reading workload [21].

A positive attitude toward the added value of AIA might change once incorporated in practice. Cè et al. [22] found that while most radiologists not using AI expect it to increase efficiency, only one‐third of those already using AIA consider it to make a decisive contribution. This contrast is confirmed by the varying distribution in answers on the statements: *AI will improve appropriate referrals during the second trimester anomaly scans; AI will reduce the number of referrals; I would struggle to refer a patient if AI suggests it but I think the scan looks normal*. Referral behavior among sonographers was previously studied by Oosterhuis et al. [23], in which *repeated visualization of an abnormal image* was the strongest indication for sonographers to refer a patient. Subtle anatomical abnormalities or minor deviations from normal anatomy, often observed for example in heart defects, may be difficult to demonstrate repeatedly within a single scan. This was hypothesized by Oosterhuis as a possible reason for missed heart defects. When AIA is also flagged during such an exam, it might serve as an additional trigger for the sonographer, even when the findings are subtle or inconclusive for the human eye.

Previous studies have shown that one of the most significant drivers of AI implementation is the high expectations regarding the potential added value of AI, including its ability to reduce diagnostic errors and enhance efficiency [10, 24]. The positive feedback from participants indicates readiness for an AI‐assisted study in a clinical setting. However, the sonographers' strong preference to have the final say in interpreting pathological findings and determining subsequent referrals may impact the clinical success of the add‐on AI tool. Will the sonographer refer if the AI tool flags an abnormality they do not recognize? Or will a flag of the AI tool provide the final nudge to refer when the sonographer hesitates to refer? In fewer than 50% of cases, sonographers consider “uncertainty” during a standard anomaly scan as a sufficient indication for referral [23]. They stated that they needed a clear abnormal image to make the decision to refer. When implementing an AI tool in clinical practice, the sonographers' influence on the referral process should therefore be considered and studied. As seen in radiologists, opinions may change during actual use [22]. Ongoing evaluation of attitudes, acceptance and behavioral effects, both during and after implementation, is essential once AI technologies are integrated into practice. In addition, qualitative research is necessary to gain a deeper understanding of expectations, barriers and challenges. Moreover, further studies should investigate patients' perspectives as well.

For this study, we were able to reach sonographers from various types of obstetric clinics across different regions of our country. Additionally, participants represented a range of training and age categories. This diversity provides a comprehensive view of the attitudes of the average obstetric sonographer in the Netherlands.

In the Netherlands, the second‐trimester anomaly scan is regulated in a nationally coordinated screening program governed by stringent quality and regulatory standards. The integration of AI into this framework would be subject to the same rigorous oversight, potentially enhancing sonographers' confidence in its safety and reliability. However, it remains uncertain whether this trust would be equally shared by sonographers in countries with less stringent regulatory standards.

It is possible that the sonographers interested in AI were more prone to answer the questionnaire than others. However, the experience with AIA in clinical practice varied widely within the study group, again suggesting that our study reflects the opinion of a diverse group of sonographers who perform anomaly scans.

## 5 Conclusion

The majority (69%) of obstetric sonographers surveyed in this study hold a positive attitude toward AIA as an add‐on tool in the fetal screening for structural abnormalities, regardless of practitioner type, setting, or years in practice. The general expectation is that the use of AIA as a diagnostic tool in standard anomaly scans will lead to an increased detection rate of abnormalities. The respondents have mixed opinions on whether AI has the potential to reduce unnecessary referrals and sonographers have a strong preference to retain the final say in interpreting pathological findings and determining subsequent referrals. Whether this may impact the clinical success of the add‐on AI tool once incorporated in practice should be further studied.

## Funding

R. A. was funded by the National Institutes of Health (R01HL150394).

## Ethics Statement

As no patient data were obtained, this study was waived from local medical ethical review board approval. This study complies with the Declaration of Helsinki.

## Consent

The authors have nothing to report.

## Conflicts of Interest

The authors declare no conflicts of interest.

## Data Availability

The data that support the findings of this study are available from the corresponding author upon reasonable request.
